# Circadian influences on dopamine circuits of the brain: regulation of striatal rhythms of clock gene expression and implications for psychopathology and disease

**DOI:** 10.12688/f1000research.9180.1

**Published:** 2016-08-24

**Authors:** Michael Verwey, Sabine Dhir, Shimon Amir

**Affiliations:** 1 Center for Studies in Behavioural Neurobiology, FRQS Groupe de Recherche en Neurobiologie Comportementale, Concorida University, Montreal, Quebec, Canada; 2 McGill University, Montreal, Quebec, Canada

**Keywords:** circadian clock, circadian rhythms, clock gene, circadian control

## Abstract

Circadian clock proteins form an autoregulatory feedback loop that is central to the endogenous generation and transmission of daily rhythms in behavior and physiology. Increasingly, circadian rhythms in clock gene expression are being reported in diverse tissues and brain regions that lie outside of the suprachiasmatic nucleus (SCN), the master circadian clock in mammals. For many of these extra-SCN rhythms, however, the region-specific implications are still emerging. In order to gain important insights into the potential behavioral, physiological, and psychological relevance of these daily oscillations, researchers have begun to focus on describing the neurochemical, hormonal, metabolic, and epigenetic contributions to the regulation of these rhythms. This review will highlight important sites and sources of circadian control within dopaminergic and striatal circuitries of the brain and will discuss potential implications for psychopathology and disease **.** For example, rhythms in clock gene expression in the dorsal striatum are sensitive to changes in dopamine release, which has potential implications for Parkinson’s disease and drug addiction. Rhythms in the ventral striatum and limbic forebrain are sensitive to psychological and physical stressors, which may have implications for major depressive disorder. Collectively, a rich circadian tapestry has emerged that forces us to expand traditional views and to reconsider the psychopathological, behavioral, and physiological importance of these region-specific rhythms in brain areas that are not immediately linked with the regulation of circadian rhythms.

## Introduction

Circadian rhythms are observed when metabolic, psychological, and behavioral processes are modulated over the course of a day, even in the absence of environmental time cues ^1– 7^. These endogenously rhythmic processes are intimately linked with the function of circadian clocks and oscillators distributed throughout the brain and body; in mammals, they are coordinated by the master circadian clock located in the suprachiasmatic nucleus (SCN) ^8– 11^. The circadian autoregulatory feedback loop, which is composed of several core circadian clock genes, provides the molecular basis for both the generation and the output of these circadian clocks and oscillators ^12– 16^. Clock genes, and in particular the Period genes (e.g. *Per1* and *Per2*), provide important circadian markers that help to highlight putative sites of circadian control as well as identify the environmental stimuli and internal signals that influence these region- and tissue-specific rhythms ^17– 25^. This omnipresent circadian system responds to the health and behavior of an individual, reacts dynamically to environmental pressures, and could interact with the progression and severity of several diseases ^26– 28^. Therefore, we must increasingly consider biological processes, lifestyle, health, and disease in the context of this ever-broadening understanding of the circadian system.

In the current review, we begin by highlighting several sites and sources of circadian regulation. In doing so, we aim to expand the “canonical” understanding of the circadian system and highlight region-specific interactions that have implications for the regulation of metabolism, stress, and epigenetics. In the first section, we provide several examples of how these daily rhythms in clock gene expression can influence daily rhythms in behavior and physiology. Moreover, in addition to their canonical roles within the circadian clock, several clock genes are also pleiotropic transcriptional regulators. Therefore, the expression of such clock genes may also interact with a range of other “non-circadian” cellular and metabolic processes. Through these examples, we hope to lend support to the potential importance of striatal rhythms, where the links with behavior and psychopathology still remain largely speculative. In the second section, we go on to describe several examples where the circadian system influences dopamine systems of the brain and explore some recent examples in which daily rhythms in dopamine release and signaling are able to feedback to influence the daily rhythms of clock gene expression in the dorsal striatum. Finally, in the third section of this review, we build on this work and discuss the potential circadian influences as they relate to psychopathology, and the broader dopamine circuitry, with an emphasis on the nucleus accumbens (NAcc). A major goal of this review is to help researchers appreciate that daily rhythms in circadian clock gene expression can be important regulators of local brain function and encourage us all to strive towards a better understanding of these circadian and pleiotropic influences of region-specific rhythms in circadian clock gene expression.

## Sites and sources of circadian control

Light is one major environmental cue that is able to produce phase adjustments in the SCN, the master circadian clock in mammals. Specifically, a subset of intrinsically photosensitive retinal ganglion cells (ipRGCs) makes projections from the retina to the SCN and functions to entrain the daily rhythms of clock gene expression in this structure with environmental light–dark cycles ^29– 32^. One of the most important aspects in this pathway is the time of day *when* light stimulation occurs, rather than the simple absence, presence, or amount of light ^4^. For instance, light during the day will typically produce little or no change in the phase of the SCN. But light during the night will produce strong phase advances or phase delays within the SCN-based circadian clock, depending on when the nighttime light is delivered, and produce related shifts in downstream behavioral and physiological rhythms ^33^. Sometimes these light pulses can have adaptive effects that push the SCN back into synchrony with environmental cycles. But when artificial sources of light are used late at night, this can also push the SCN out of synchrony with the environment and could serve to exacerbate the same circadian disruptions that may have disrupted sleep in the first place. A growing literature has been supporting this hypothesis that light at night, acting through either the circadian clock or other light-responsive circuitry, can have negative consequences on sleep, health, mood, and disease ^34– 36^.

Daily rhythms in circadian clock gene expression have also been documented in many other tissues and nuclei of the brain and body, including the amygdala, hippocampus, oval nucleus of the bed nucleus of the stria terminalis, and olfactory bulbs ^17– 20, 23, 37– 39^. However, extra-SCN oscillations of clock gene expression are not typically entrained by light directly. Instead they utilize neural, hormonal, metabolic, and neurochemical rhythms to remain entrained with the environment and maintain an appropriate internal organization with each other ^40– 45^. The demands, reactivity, and function of each brain area and tissue are highly region specific. Likewise, the daily oscillations of circadian clock gene expression are also region specific, and each region exhibits a unique peak, trough, and phase in local oscillations of clock gene expression ^24^. As a result, we must also seek to better understand the influences on, and co-ordination between, the many extra-SCN circadian oscillators contained within the brain.

Daily oscillations in clock gene expression have been linked with specific functions in only a few cases. For instance, in the olfactory bulb, it took several years after circadian rhythms in clock gene expression were first observed ^18^ for a link to be made between these rhythms in clock gene expression and the control of olfactory responsivity ^46, 47^. Likewise, daily rhythms in circadian clock gene expression in the adrenal gland have also been shown to be an important gating mechanism in the control of daily rhythms in glucocorticoid release ^14^. In many other cases, however, the specific sites of circadian control remain unclear. For instance, changes in the light–dark cycle that simulate jet lag induce cognitive deficits that affect fear-conditioning ^48^. While this effect likely involves the amygdala and/or hippocampus, the direct link between changes in fear-conditioning and circadian clock gene expression in either of these structures remains tentative. Moreover, global mutations in individual clock genes also produce important changes in behavior, neurochemistry, and mood ^49, 50^, but, again, the site(s) of circadian control that are relevant to these effects are still being identified. Therefore, these links between site-specific clock gene expression and functional consequences in behavior and physiology continue to be another ongoing and major challenge for future research.

Metabolic processes are another major influence on, and consequence of, the circadian system ^51– 56^. In rodents, restricting food-availability to a single mealtime each day engages a network of food-entrainable oscillators in the brain ^57^. Restricted feeding is a compelling area of study because it is a rare example of an explicitly circadian phenomenon that does not rely on the SCN ^58^. However, the critical site(s) for circadian food-entrainment have remained elusive ^59– 61^ and the degree to which food-entrainment relies on circadian clock gene expression also remains unclear ^60, 62, 63^. Even so, the daily patterns of circadian clock gene expression are responsive to restricted feeding schedules, and this response is also region specific ^57^. For example, restricted feeding schedules that provide daytime or nighttime meals will adjust circadian oscillations in some overlapping brain areas, but some of these effects will also depend on the meal *time* rather than the restriction, *per se* ^42, 43^. The importance of the circadian time when food is consumed is further emphasized when rodents are fed a high-fat diet during either their active or their inactive period ^64^. Such simple changes in the time of food availability/consumption can exacerbate or mitigate weight gain, even if the number of calories consumed does not change. The description of these complex interactions between the metabolic and circadian systems continues to be another major interest in the field. A better understanding of this interaction will highlight a secondary pathway, which, in addition to light, could be used to suggest appropriate mealtimes that produce adaptive adjustments within the circadian system and help to “normalize” rhythm disruptions linked to jet lag, shift-work, or psychopathology.

Extra-SCN rhythms in clock gene expression integrate state variables and, depending on the tissue, can respond to a wide variety of environmental and hormonal cues. For example, the hypothalamic-pituitary-adrenal (HPA) axis provides an interesting example of interaction between the systems that regulate circadian rhythms and stress. There is a strong daily rhythm in the release of glucocorticoids from the adrenal that depends on the SCN ^8^. Subsequently, these rhythms in glucocorticoid release are also crucial to the entrainment and maintenance of circadian rhythms in clock gene expression in parts of the limbic forebrain and amygdala ^41, 65, 66^. Acute stressors, which produce a short-term peak in glucocorticoid release, also produce acute changes in the expression of certain clock genes in many of the same regions ^67, 68^. Therefore, in diseases like depression, where baseline and stress-reactive changes in glucocorticoid release occur, it is becoming increasingly important to also consider the potential downstream effects on the rhythms of clock gene expression in the brain.

An emerging relationship also exists between circadian rhythms and the field of epigenetics. Epigenetics broadly refers to the study of modifications to the chromatin structure of DNA, which help to regulate gene expression without altering the underlying genetic code. Chromatin is composed of DNA wrapped around an octamer of histone proteins that form the nucleosome. Post-translational modifications to the N-terminal tails of histones are involved in diverse biological processes such as transcriptional activation and inactivation, chromosome packaging, and DNA damage and repair ^69, 70^. The histone modifications that produce these diverse effects include acetylation, methylation, phosphorylation, and ubiquitination. In addition to acting within the circadian autoregulatory feedback loop, clock proteins such as CLOCK, PERIOD (PER), and CRYPTOCHROME (CRY) also facilitate some of these epigenetic processes ^71^. Specifically, CLOCK acts within the canonical circadian feedback loop, but it is also a histone acetyltransferase ^72^. Acetylation of the histone tail neutralizes the positive charge and renders the chromatin more “open”. This conformational state is associated with active transcription and gene expression ^73^ and is facilitated by histone acetyltransferases such as CLOCK. Moreover, PER and CRY provide negative feedback within the circadian loop and, as it turns out, also recruit SIN3A/SIN3B and go on to associate with histone deacetylases that remove acetyl groups from the histone tails and limit gene transcription ^74, 75^. The time course of these epigenetic effects remains unclear but is possibly relevant for hour-to-hour changes that are observed across the 24-hour cycle. Moreover, in conditions where clock genes are mutated or where single-nucleotide polymorphisms create lasting changes in circadian clock protein function, the epigenetic consequences could have additional life-long or even trans-generational consequences.

Epigenetic effects also lie at the interface between metabolic and circadian rhythms ^71^. In particular, nicotinamide adenine dinucleotide (NAD ^+^) is involved in reduction–oxidation reactions, which are key to metabolism at the cellular level and are rhythmic over 24 hours ^76^. As a result of these daily rhythms, NAD ^+^ is ideally positioned to respond to restricted feeding schedules that limit food-availability to a single meal each day. Downstream of this response, NAD ^+^ is also a cofactor for SIRTUIN (SIRT) proteins, which are also histone deacetylases ^77– 79^. This epigenetic pathway is a compelling possibility for how restricted feeding schedules feedback onto and modulate diverse clock-controlled processes. These metabolic pathways may also be differentially important depending on which brain area or tissue is examined. Consistent with this idea, the shifts that are observed in the daily oscillations of clock gene expression produced by restricted feeding schedules are typically region specific ^42, 43, 57^. As a result, such entrainment mechanisms would appear to interact and “summate” with the other hormonal and neurochemical signals, which also influence clock gene expression in a region-specific manner outside of the SCN ^45, 66^. Collectively, these findings point to a distributed response, which influences several brain areas, interacts with many biological systems, and helps to highlight several brain areas that are sensitive to the effects of feeding.

## Dopamine, drugs, and disease

The dopamine systems of the brain have been implicated in many aspects of behavior, from fundamental motor control and endocrine release to higher-order processing like prediction error and attention ^80^. Dopamine systems also interact with the expression of circadian clock genes. For instance, in the retina, dopamine synthesis and receptors are adjusted in response to light, and dopamine signaling goes on to influence the expression of clock genes ^81– 83^. There is also a growing interest in the potential interactions between dopamine systems and circadian rhythms in the brain ^84, 85^. Drugs of abuse such as cocaine and amphetamine act, in part, through the dopamine system to increase locomotor activity in rodents. When PER1- and PER2-mutant mice are tested, the acute effects of these drugs can be quite similar between mutants and wild-type controls, but locomotor sensitization is fundamentally changed in the mutant mice ^49^. Studies have also shown that drugs of abuse can also induce the expression of several circadian clock genes in the dorsal striatum ^86^, which demonstrates another reciprocal interaction between these two systems. While these initial findings supported the general hypothesis that certain clock-related processes can influence dopamine plasticity and function, they did not determine the sites of action or the directionality of these effects. Therefore, a major goal in this area has been to uncover the brain areas and the molecular processes underlying these interactions.

One area of overlap between these two systems is found in the demonstration that the expression of enzymes integral to the production and metabolism of dopamine is influenced by circadian clock genes. In particular, an enzyme that is involved in dopamine metabolism, monoamine oxidase A (MAOa), is clock controlled and influences mood-related behaviors ^87^. Likewise, the rate-limiting enzyme in the production of dopamine, tyrosine hydroxylase (TH), is also regulated by the circadian clock ^50, 88, 89^. Beyond the synthesis and metabolism, dopamine release also goes on to provide a robust circadian drive that makes important contributions to rhythms in behavior and physiology. For instance, melatonin release from the pineal gland is highly rhythmic and often used as a circadian marker in human studies. The release of melatonin also relies on the heteromerization of adrenergic receptors with dopamine D4-receptors, which positions dopamine as an important regulator of pineal function ^90^. Likewise, daily rhythms in dopamine have also been observed in other hypothalamic neuroendocrine neurons, which control the precisely timed release of reproductive hormones such as prolactin. These dopamine neuroendocrine neurons also express circadian clock genes, which provide some of the circadian regulation within this system ^91, 92^. So, depending on where we look in the brain, dopamine could be an important input to the daily rhythms of circadian clock gene expression, or dopamine could also function as an important daily cue that goes on to provide a diurnal/circadian signal to other brain areas or neural systems.

Dopamine cell bodies in the ventral tegmental area (VTA) and substantia nigra (SN) of the ventral midbrain hold tremendous importance for motivation and emotion. Dopamine cell bodies in the VTA make several distinct projections and provide major dopamine input to the ventral striatum, cortex, and limbic system. In contrast, dopamine cell bodies in the SN are best known for their roles in motor functions and project mainly to the dorsal striatum ^93^. Because of their profound behavioral relevance, there has also been a growing interest in understanding the behavioral and neurochemical implications of the circadian clock(s) related to these circuits ^94^. In particular, dopamine release provides a robustly rhythmic signal in both the dorsal and the ventral striatum ^95^. Specifically in nocturnal rats, during the night when these rodents are most active, extracellular dopamine levels in the striatum are elevated ^45, 96, 97^. Daily changes in dopamine production, transport, reuptake, and metabolism could also help to increase the amplitude of daily rhythms within this system ^95, 98^. Daily rhythms in dopamine release within this circuitry also produce circadian rhythms in several electrophysiological parameters such as local field potential, firing frequency, and coherence ^99^. Therefore, daily rhythms in dopamine release from VTA and SN projections to the dorsal and ventral striatum could provide an important daily drive that goes on to influence motivation, emotion, and ultimately behavior ^94^.

Robust daily rhythms in *PER2* expression are also observed in both the dorsal and the ventral striatum ^24, 37, 100– 102^. Therefore, we and others have become interested in describing the importance of dopamine for the maintenance, entrainment, and generation of rhythms in clock gene expression in the striatum ^45^. To this end, we have used unilateral injections of 6-hydroxydopamine (6-OHDA) in the medial forebrain bundle to lesion dopamine projections on one side of the brain and study the effects on daily rhythms of *PER2* expression in the dorsal striatum. Compared to the devastating behavioral effects of bilateral lesions, unilateral lesions allow the rodents to remain healthy. These rodents continue to exhibit relatively normal rhythmicity in many aspects of behavior and physiology ^103^, but dopamine denervation causes a significant decrease in the amplitude of the rhythm of *PER2* expression on the lesioned side ^45^. After these initial observations, we went on to use systemic administration of pharmacological dopamine receptor antagonists or agonists to describe the receptor specificity of *PER2* expression rhythms in the dorsal striatum. Under *ad libitum* feeding conditions, we found that dopamine appeared to be acting through D2-receptors to synchronize *PER2* expression rhythms in the medium spiny neurons of the dorsal striatum ^45^. However, another study recently used D1-receptor knockout mice under restricted feeding conditions and found blunted *PER2* expression rhythms in the dorsal striatum of mutant mice ^104^. These findings suggest that the D1 and D2 specificity may not be as clear as the pharmacology initially indicated or that these effects may also depend on feeding conditions. A D2-receptor-specific effect would be consistent with other observations in the striatum and retina ^82, 83, 105, 106^ and could point to a more general pathway that links dopamine signaling with effects on circadian clock gene expression. However, because robust dopamine receptor antibodies are largely unavailable, moving forward, we must rely on genetic approaches that can reliably differentiate D1- and D2-receptor-containing neurons in the striatum.

The amplitude of the daily rhythm in dopamine release from VTA and SN terminal regions may also contribute to the amplitude of rhythms in clock gene expression ^45, 96, 97^. We have already shown that dopamine release and clock gene expression is intimately linked in the dorsal striatum ^45^. The amplitude of this rhythm, however, could be a major factor driving clock gene expression within the ventral striatum as well. In line with this hypothesis, large amplitude rhythms in dopamine release are observed in the NAcc core, while smaller amplitude rhythms are observed in the shell ^95^. Likewise, large amplitude rhythms in *PER2* expression are observed in the NAcc core, while smaller amplitude rhythms are observed in the shell ^24^. One could therefore speculate that the rhythmic release of dopamine may also serve as an important driver of circadian clock gene expression throughout both the dorsal and the ventral striatum.

The ability of dopamine to cause changes in clock gene expression could also have a number of important implications for disease severity and treatment. For instance, in conditions such as Parkinson’s disease, dopamine projections to the dorsal striatum degenerate. Reduced dopamine input would then likely blunt daily rhythms in clock gene expression in the striatum of these patients and could be an important consideration in the etiology of circadian disruptions linked with this disorder ^84^. Moreover, drug treatments that produce a surge of dopamine release, such as methamphetamine or cocaine, also produce alterations in the expression of several core circadian clock genes in both the dorsal and the ventral striatum ^37, 107– 110^. This general principle could also be applied to virtually any prescription drug treatment that is given systemically and which acts as an agonist or antagonist of dopamine signaling. For example, methylphenidate is used in the treatment of attention deficit hyperactivity disorder and produces elevated dopamine release. This surge in dopamine release could go on to influence the daily rhythms of clock gene expression for hours or days, long after the drug itself has worn off. Conversely, several antipsychotics used in the treatment of schizophrenia have historically antagonized dopamine receptors, which could also blunt striatal rhythms in clock gene expression. In summary, the broadening tapestry of circadian control could be implicated in a wide range of disorders, and these circadian considerations would seem to highlight several potential benefits and/or pitfalls of many treatment options.

Diverse and reciprocal effects of dopamine on the circadian system and the circadian system on dopamine still leave the causal relationship(s) between these two systems unclear. Novel therapeutic avenues could be raised through a better understanding of these interactions, which could go on to help patients improve nighttime sleep, improve daytime alertness, help reduce symptoms, or help confine symptoms to a time of day when they are more tolerable. Such chronotherapeutic improvements will probably end up being the result of careful timing of prescription pharmaceuticals in conjunction with the appropriate management and timing of other environmental stimuli (e.g. light and food). A better understanding of the times of day when symptoms are consistently better or worse may also be able to guide patients towards an improved general understanding of their own conditions.

## Psychopathology

The general links between disrupted circadian rhythms and the symptoms of psychopathology have been discussed for some time. Indeed, major depressive disorder, bipolar disorder, and seasonal affective disorder all exhibit links with certain polymorphisms in clock genes ^111– 113^. Recently, however, specific brain areas that are relevant to the circadian control of psychopathological symptoms have also started to be uncovered. Generally, stressful stimuli can be used to induce depression-like phenotypes in rodents, and this has provided several interesting models to study depression. In one such animal model of depression, unpredictable chronic mild stress was shown to change the amplitude of rhythms of clock gene expression in the NAcc ^25^. Importantly, the severity of this blunting of NAcc rhythms was correlated with the severity of depression-like behaviors in these same mice, and this has provided a compelling link between circadian rhythms in the NAcc, stressors, and symptom severity within this model of depression.

Circadian rhythms in the NAcc have also been shown to be affected in another model of depression, which uses learned helplessness to induce a depressive phenotype in rodents. Remarkably, this model also produces blunted circadian rhythms in the NAcc ^114^, which provides converging evidence for the link between symptoms of depression and rhythms within the NAcc. However, it is not yet known whether changes in circadian rhythm are a cause or consequence of stress or depression. In addition, both groups have also reported changes in the amplitude of clock gene expression within the SCN, which were also associated with depression-related behaviors ^25, 115^, and it is another major challenge to dissociate SCN and extra-SCN effects. However, the ability to induce site-specific disruptions of circadian clock gene expression may eventually help to highlight and differentiate SCN from extra-SCN circadian contributions to psychopathology, and we look forward to studies that disrupt the expression of circadian clock genes selectively within the dorsal or ventral striatum.

Other brain areas could also make direct and indirect contributions to activity within these dopamine circuits. For many years, we have known that the habenula can inhibit the activity of dopamine neurons in the VTA and SN ^116^. The habenula also exhibits daily oscillations in neuronal firing, and certain cells either fire or suppress firing in response to environmental light ^117, 118^. Remarkably, a more recent paper has shown that the lateral habenula also responds to learned helplessness ^119^, the same model of depression used by Landgraf *et al*. Therefore, because the habenula receives inputs from midbrain dopamine structures ^120^ and provides feedback to the VTA ^121^, we suggest that it could also provide important diurnal influences on the VTA. Another source of circadian regulation of dopamine circuitry also comes from the medial prefrontal cortex (mPFC). When the mPFC is lesioned, rhythms of cFos immunoreactivity in the NAcc are severely blunted without affecting daily rhythms in cFos immunoreactivity in the VTA ^122^. Inactivation experiments have shown that the mPFC can also blunt the diurnal rhythm in amphetamine reward ^123^, suggesting that the mPFC can modulate the circadian properties of this dopamine circuitry. Thus, in addition to endogenous rhythmicity and clock gene expression within the NAcc, there are other circadian influences from the mPFC as well as light-responsive and stress-responsive rhythms from the lateral habenula that should also be considered. As a result, we must continue to strive to consider the behavioral implications at a network level rather than focusing too much on any single node.

## Conclusions

It is a major challenge to describe interactions within the broadening context of the circadian system, which contains multiple loci of control and feedback. The effects of glucocorticoids, for instance, could represent an important interface between circadian regulation and psychopathology. The effects of neurotransmitters such as dopamine on clock gene expression provide another level of analysis, which could link diseases and disorders with circadian disruptions and symptoms. Region-specific rhythms of clock gene expression are sensitive to hormonal and neurochemical controls, but this also varies from region to region. The importance of these interactions between circadian systems and health is further emphasized by the finding that frequent changes in the light–dark cycle challenge the circadian system and have been shown to shorten lifespan ^124^. In order to advance our understanding of the implications for health and disease, we must first encourage all researchers to consider the circadian influences within their own given areas of expertise. It is no longer enough to simply control for the time of day; instead, we should each strive to understand how these daily fluctuations are influencing research and findings in our respective areas.
